# Mapping urban form into local climate zones for the continental US from 1986–2020

**DOI:** 10.1038/s41597-024-03042-4

**Published:** 2024-02-13

**Authors:** Meng Qi, Chunxue Xu, Wenwen Zhang, Matthias Demuzere, Perry Hystad, Tianjun Lu, Peter James, Benjamin Bechtel, Steve Hankey

**Affiliations:** 1 School of Public and International Affairs, Virginia Polytechnic Institute and State University, Blacksburg, VA 24060 USA; 2 College of Earth, Ocean, and Atmospheric Sciences, Oregon State University, Corvallis, OR 97331 USA; 3 Edward J. Bloustein School of Planning and Public Policy, Rutgers University, New Brunswick, New Jersey 08901 USA; 4 Urban Climatology Group, Department of Geography, Ruhr-University Bochum, Bochum, 44801 Germany; 5 College of Health, Oregon State University, Corvallis, OR 97331 USA; 6 Department of Epidemiology and Environmental Health, University of Kentucky, Lexington, KY 40536 USA; 7 Department of Population Medicine, Harvard Medical School and Harvard Pilgrim Health Care Institute, Boston, MA 02215 USA; 8 Department of Environmental Health, Harvard T.H. Chan School of Public Health, Boston, MA 02215 USA

**Keywords:** Environmental impact, Research data

## Abstract

Urbanization has altered land surface properties driving changes in micro-climates. Urban form influences people’s activities, environmental exposures, and health. Developing detailed and unified longitudinal measures of urban form is essential to quantify these relationships. Local Climate Zones [LCZ] are a culturally-neutral urban form classification scheme. To date, longitudinal LCZ maps at large scales (i.e., national, continental, or global) are not available. We developed an approach to map LCZs for the continental US from 1986 to 2020 at 100 m spatial resolution. We developed lightweight contextual random forest models using a hybrid model development pipeline that leveraged crowdsourced and expert labeling and cloud-enabled modeling – an approach that could be generalized to other countries and continents. Our model achieved good performance: 0.76 overall accuracy (0.55–0.96 class-wise F1 scores). To our knowledge, this is the first high-resolution, longitudinal LCZ map for the continental US. Our work may be useful for a variety of fields including earth system science, urban planning, and public health.

## Background & Summary

The global population is increasingly shifting to urban areas (i.e., 56% of the population currently lives in urban areas and is projected to increase to 68% by 2050)^1^. While much of the future urbanization is expected to occur in Asia and Africa, studying historical urban development patterns for areas that have already experienced high degrees of urbanization can provide valuable data to inform global urban planning and growth management efforts. Cities will be a key component of solutions that aim to achieve sustainable development goals since they are home to large populations that influence a large share of total energy consumption and anthropogenic emissions^2,3^. Targeted urban planning and growth management will be crucial for sustainable development in every dimension, for example, economic growth, societal and political well-being, public health, and environmental sustainability^4–8^.

A fundamental requirement for studying the historic impact of urban development and expansion is a reliable longitudinal dataset that measures urban form and urban functions. Urban form is defined as the physical characteristics of built-up areas (at various scales), including building density, building heights, construction materials, street layouts, and fraction of green space^9,10^. Urban functions are made possible by various infrastructure systems from diverse sectors (e.g., energy, transport, waste management, etc.). As a holistic system, the interplay of urban form and urban functions shapes the built and natural environment, which in turn influences people’s daily activities, exposures to environmental pollutants (e.g., air pollution, heat flux, hazardous materials), and impacts to human health^11,12^. Urbanization also drives local and regional changes in climate, leading to phenomena such as the urban heat island [UHI] and urban dry island [UDI] effects^3,13,14^. The UHI effect (i.e., the phenomenon of relatively higher temperatures in urban areas compared to surrounding non-urban areas) is an emerging risk factor for urban residents and has been extensively studied^15–21^.Along with global climate change, urban residents are expected to face more frequent extreme heat events and exacerbated heat stress, which poses significant risks to human health and well-being^22–24^.

In urban climate science, urban datasets that provide key parameters of the urban environment are crucial as they are a required component for urban climate and weather modeling. Datasets that only report simplified urban versus nonurban or a low level of thematic classification limit urban and climate analyses. For example, in-depth UHI analysis by different urban and natural forms necessitates a more detailed urban form classification. In addition, some empirical urban parameterizations derived for specific cities or regions may not transfer to other cities, regions or generalize to larger scales (e.g., continental or global). A detailed and harmonized urban classification scheme tailored to climate impacts is a critical need for urban studies. Driven by this need, Stewart and Oke proposed a generic, culturally-neutral urban form classification scheme called Local Climate Zones [LCZ] which is meant to be local in scale, climatic in nature, and zonal in representation^25^. The LCZ scheme consists of 10 built classes and 7 land cover classes and was originally designed to provide field metadata for UHI observational studies^25,26^. Stewart and Oke estimated and provided representative physical characteristics of each LCZ category including geometric and surface cover properties (e.g., sky view factor, impervious/pervious surface faction, roughness), thermal, radiative and metabolic properties (e.g., surface albedo, anthropogenic heat output) – all of which can play a key role in urban climate modeling^25^. The scheme is generic and thus considered to be an urban classification solution that is applicable worldwide. A number of studies have shown improved model performance for urban climate modeling after using urban parameters derived from LCZ datasets^27–30^.

Due to these advantages, applications of the LCZ concept have become increasingly common^31–34^. In 2015, the World Urban Database and Access Portal Tools [WUDAPT] project initiated a community-based effort to collect global LCZ training data and proposed a straightforward LCZ mapping workflow that relies on freely available remote sensing data, crowdsourced training areas, and open software for supervised LCZ classification^35,36^. The WUDAPT project facilitated a remarkable growth in remote sensing based LCZ mapping studies – of which most are developed at the city and regional scale^34,37–42^. Also, the LCZ Generator^43^ recently contributed to a significant increase in city-level LCZ maps. Time-series LCZ maps are available at the city or regional scale for a small number of locations (e.g., Bangkok in Thailand, Kunming and Pearl River Delta in China) for a few specific years^44–46^. In contrast, only a limited number of LCZ maps have been generated at the national (i.e., USA^47^), continental (i.e., Europe^48^), and global scale^9,49^. Many of these efforts have been aided by Google Earth Engine [GEE], a cloud-based platform that provides remote sensing datasets at the petabyte-scale and powerful online cloud-computing capabilities^34,50^. A main limitation of these existing large-scale maps is that they are for a single year. To date, no longitudinal LCZ maps are available at national, continental or global scales. The lack of large-scale time-series LCZ maps represents a significant gap in the current LCZ literature. Addressing this gap holds importance to the scientific community interested in exploring the impacts of the growth and change in urban environments overtime.

In this study, we introduce longitudinal LCZ maps with fine spatial resolution (100 meters) for the continental US [CONUS] from 1986 to 2020. Our modeling pipeline generally followed WUDAPT’s protocol but was modified to allow large scale mapping with temporal consistency across years. We developed contextual random forest [RF] classifiers with hybrid model development, including (1) crowdsourced and expert training area labeling, (2) local model fine-tuning and cloud model reproduction, and (3) large-scale LCZ surface mapping and post-classification processing. We incorporated publicly available Landsat imagery as well as existing land use and land cover [LULC] datasets and Census information to further improve our model accuracy. To our knowledge, this is the first LCZ dataset that spans over 35 years for the continental US. We anticipate that this dataset will aid future studies with a range of potential applications, e.g., UHI studies, urban climate and weather modeling, urban expansion, etc.

## Methods

**Fig. 1:**
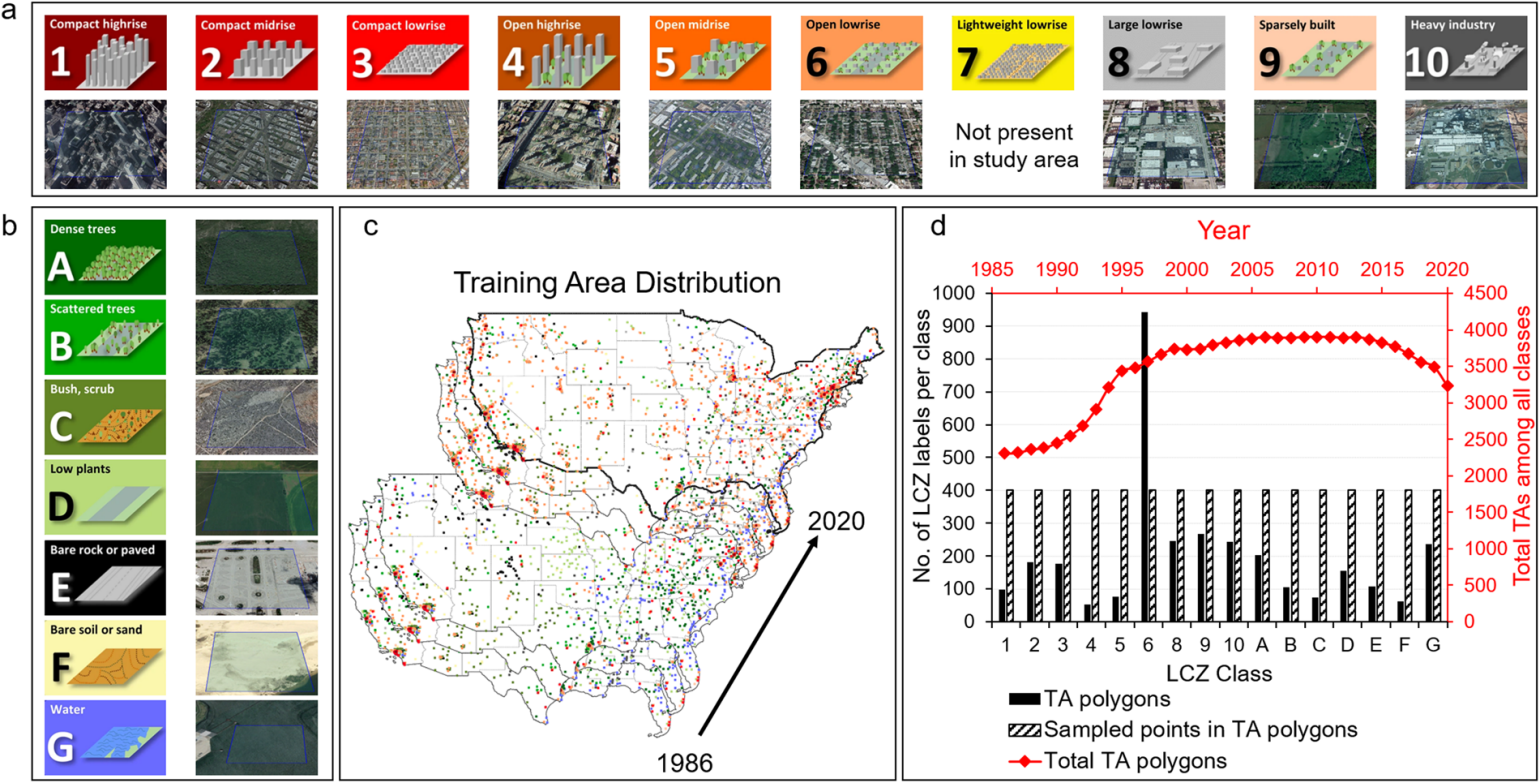
LCZ classification scheme including (**a**) 10 built classes (i.e., LCZs 1–10) and (**b**) 7 land cover classes (i.e., LCZs A-G). Panels (**a**) and (**b**) are adapted from Fig. 1 in Demuzere *et al*.^47^; representative aerial maps (© Google Earth 2020) for each LCZ type are shown. (**c**) The spatial-temporal distribution of LCZ TAs across CONUS. The colors of TAs are consistent with the color palette presented in panels (**a**) and (**b**). (**d**) Counts of (1) total TAs by year (red line), (2) TAs by LCZ class in 2020 (black solid bars), and (3) sampled points within TA polygons by class for each year (black stripe bars) for model development.

The geographic focus of our study is CONUS from 1986 to 2020. Training Areas [TAs] were collected for each year distributed across CONUS for 16 LCZ classes (Fig. 1). Following previous work^47^, we did not include LCZ 7 (lightweight lowrise) in our model since it rarely occurred in our study area. Our hybrid modeling pipeline included (1) hybrid TA labeling, (2) cloud-enabled RF classifier development, and (3) model prediction and post-classification processing. After model development and prediction, we used a number of approaches to evaluate model performance, including 5-fold spatial cross validation for accuracy evaluation, benchmarking against external land use and LCZ maps, and an urban sprawl analysis based on our LCZ product.

### Hybrid TA labeling

Our approach to collect TAs included two steps. First, we leveraged the labeling power of a crowdsourcing platform – Amazon Mechanical Turk [MTurk] (https://www.mturk.com/) following the work of Demuzere *et al*.^47^ Briefly, we sampled 500 m × 500 m TAs across CONUS. For each area, we showed 10 unique MTurk workers the corresponding satellite and aerial image from Google Earth (https://www.google.com/earth/) and street-level images from Google Street View (https://www.google.com/streetview/). The MTurk workers voted for the LCZ category that each TA best represented. Only TAs with at least 70% agreement among the 10 MTurk workers were considered as valid crowdsourced labels. Details on this labeling approach can be found in Demuzere *et al*.^47^ The base year for the crowdsourced training labels is 2017 since our TAs expanded on the effort from Demuzere *et al*.^47^ We further collected TAs across CONUS by randomly sampling from an existing LCZ map^47^ for each LCZ category to ensure balance across LCZ types in our training data. For example, to sample more TAs for LCZ 1, we randomly sampled from all pixels that were classified as LCZ 1 in the LCZ map representative for year 2017^47^, created corresponding 500 m × 500 m polygons and sent them to MTurk to follow the approach above. To improve the balance among all LCZ categories, after requiring 70% agreement among MTurk workers, we manually checked satellite imagery on Google Earth to add additional TAs for under-sampled LCZs.

To expand the temporal coverage of TAs from 1986 to 2020, we developed a manual labeling procedure to add temporal labels to the single year crowdsourced labels. Specifically, we manually examined the valid MTurk labels and added labels for other years by comparing historical satellite imagery using the Google Earth time slider and assessing when the land cover or urban form changed (e.g., due to development). The imagery in Google Earth was collected through various data providers and platforms. Hence, the temporal and spatial resolution of historical imagery available in Google Earth varies largely depending on the source data and TA location. Typically, less historical imagery is available in early years due to the limited availability of satellite imagery sources. We used three steps to ensure the quality of the TAs. First, when the expert’s opinion differed from the consensus of MTurk votes (i.e., over 70% of agreement), the experts would provide their classification as well as their confidence level (high/medium/low) in that classification. Among 4,422 valid MTurk TAs, the expert agreed with 83% of classifications for the base year and marked them as high confidence. Second, when examining historical satellite imagery for each TA, if the land cover or urban form changed significantly and the expert believed that the LCZ category should be changed to another LCZ type, they either stopped historical labeling for that TA to ensure accuracy of the TA or labelled it to another LCZ class with a confidence level. Third, we only considered TAs that had high confidence for model development. TA labels from 1986–2020 were compiled after the hybrid labeling process. Due to the availability of historical aerial maps in early years (e.g., gaps during some early years from Google Earth) and our approach to stop historical labeling when the TA substantially changed, fewer TAs were available in early years (e.g., 1986–1995) for model development (Fig. 1d). This effect is also apparent (although less so) for TAs during 2017–2020 mainly due to changes (e.g., development) that happened after the base year.

### Cloud-enabled model development

**Fig. 2:**
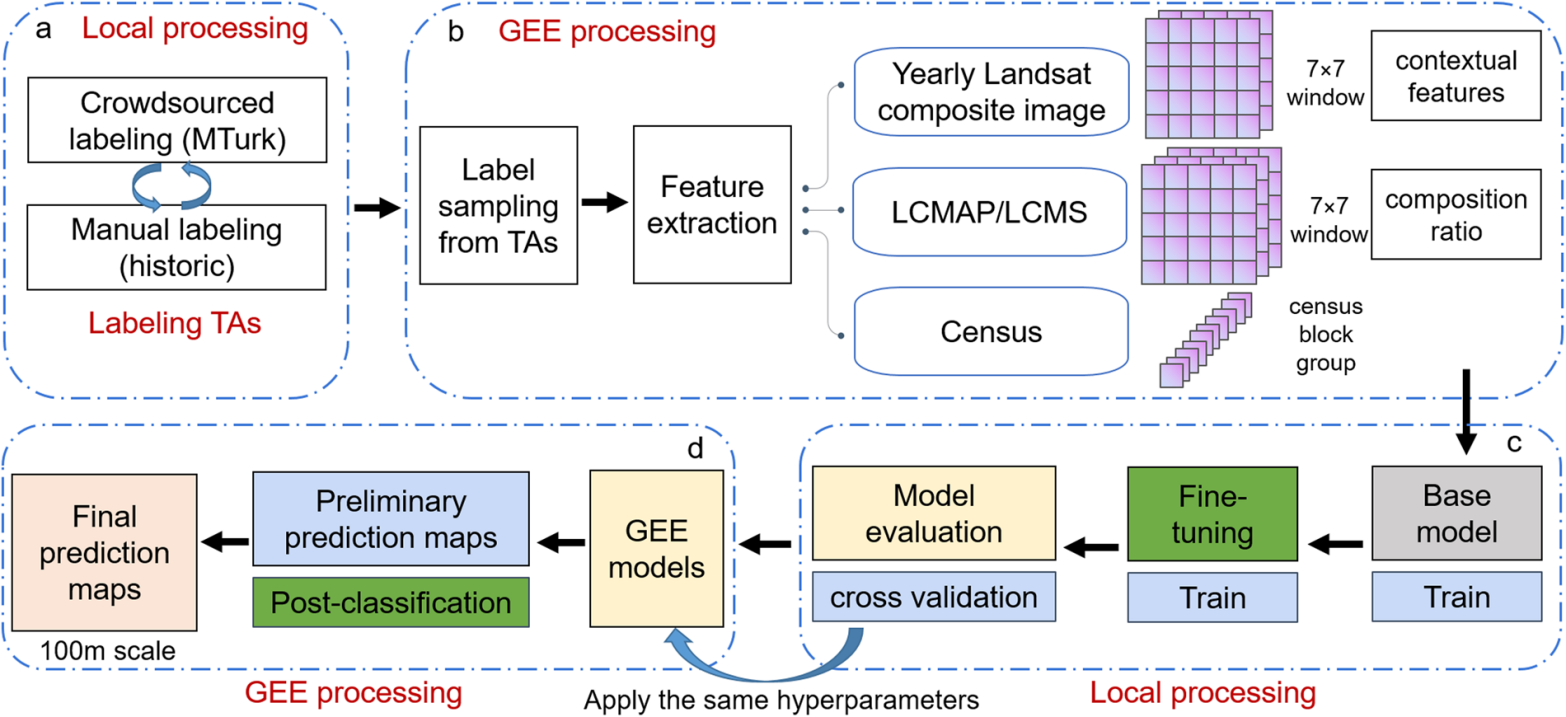
Model development workflow. (**a**) TA polygon labeling including crowdsourced labeling using the MTurk platform for the base year (2017) and manual labeling by experts for other years (1986–2020). (**b**) Balanced label sampling from TA polygons and model feature extraction including features collected from yearly Landsat composite layers, LULC layers (LCMAP and LCMS), and Census layers. (**c**) Model training, fine-tuning, and evaluation on a local machine using Python scikit-learn library. (**d**) Model transfer from the local machine to the GEE platform, post-classification processing, and final LCZ prediction surfaces at 100 m × 100 m resolution from 1986–2020 across CONUS.

The WUDAPT protocol uses a pixel-based random forest modeling approach^51^. Other work showed that a contextual RF classifier, which incorporates neighborhood information, can significantly increase model accuracy (e.g., as high as 13% increase in overall accuracy)^52^. Some recent studies developed and applied more advanced algorithms such as convolutional neural networks [CNN] and recurrent residual network [Re-ResNet] for LCZ classification^49,53–56^. Algorithm comparisons have shown that CNN can significantly outperform RF^55,56^. For example, Rosentreter *et al*. compared CNN, pixel-based RF, and contextual RF for LCZ classification in multiple cities. In general, CNN achieved the highest model performance, with an average increase in overall accuracy of 16.5% and 4.8% compared to pixel-based RF and contextual RF^55^. Yoo *et al*., found that the improvement of model performance by incorporating the neighboring area of a focus pixel is significant, not only among RF models but also among CNN models^56^. However, deep learning algorithms require more intense computation resources than lightweight RF models. Our preliminary analyses showed that applying a deep learning model is still a difficult task for up-scaling to our target scale (i.e., national scale over 35 years) even with the aid of the cloud-computing GEE platform. For example, CNN requires input images with high enough resolution to achieve deep layers due to the convolution, pooling or drop out techniques. Using 10 m satellite images (a higher resolution would be even better) for instance, our preliminary analyses showed that the image file size for a single year would exceed two Terabytes, requiring huge computation resources for image processing and model application. In our study, we aim to develop lightweight contextual RF models for large-scale time-series LCZ mapping based on a hybrid model development pipeline (Fig. 2). The choice of the lightweight contextual RF algorithm as well as the local-cloud hybrid modeling pipeline were mainly driven by the feasibility for large scale LCZ mapping.

Since an imbalanced training set generally hinders the performance of a machine learning model, we adjusted our label sampling strategy accordingly. As shown in Fig. 1d, the number of TAs collected during labeling varied among LCZ types. For example, we obtained far more LCZ 6 (open lowrise) as compared to other categories and few TAs for LCZ 4 (open highrise) and LCZ F (bare soil or sand). To assemble a balanced training dataset for model development, we undersampled from LCZ 6 TAs and oversampled from other LCZ TAs so that each year and each LCZ category had the same amount of training samples (i.e., 400 sample points per year per class). In addition, to improve model performance, we only sampled from a 200 m × 200 m area near the centroid of the TA polygons instead of the entire 500 m × 500 m polygon based on our sensitivity analysis (see Supplementary Information: **Sensitivity analysis for sampling regions** and Table S1).

After sampling from TA polygons, we extracted model predictors through the GEE platform (Fig. 2b). Due to the lengthy study period (i.e., 1986–2020), only a limited number of datasets are available for extracting predictor features over the entire period. Our models used three types of input feature layers: (1) yearly Landsat composite imagery, (2) existing yearly LULC layers including LCMAP (Land Change Monitoring, Assessment, and Projection)^57^ and LCMS (Landscape Change Monitoring System)^58^, and (3) Census data (i.e., total population and population density at the Census Block Group level)^59^. The Landsat composite imagery was derived from Landsat imagery datasets retrieved via GEE platform^50^. We also included year as a dummy variable. For Landsat variables, we extracted features from 12 bands, including 7 Landsat bands and 5 derived spectral indices, such as BCI (Biophysical Composition Index) and NDBAI (Normalized Difference BAreness Index)^47^. We also included 16 land cover bands from LCMAP and 22 land cover/land use bands from LCMS. More details can be found in Supplementary Information Table S2. For our contextual RF models, we collected contextual features for both Landsat and LULC features. Specifically, we collected features within a 7 × 7 window for each sampling point, which was equivalent to a 210 m × 210 m neighboring area patch since both Landsat and LULC layers have a 30 m spatial resolution. For the Landsat variable, we collected 6 statistics of the input features within the sampling window: mean, max, min, median, the 25^th^ percentile and the 75^th^ percentile. For LCMAP and LCMS, we calculated the composition ratio for each land cover/land use type within the sampling window. For the Census layer, we directly collected the census values at the locations of samples given that the census block group size varies across regions. We also tested the introduction of another type of census data (i.e., segmented employment data) into the model. However, employment data was only available after 2002 thus was not considered for our final pipeline. Despite this, we report the details in Supplementary Information (**Incorporating segmented employment data into the LCZ model** and Fig. S1) as employment data helped in distinguishing between certain urban classes and may be useful for future efforts.

After assembling the training set, we conducted two modeling steps: (1) fine-tuning a RF model on a local machine using the Python scikit-learn library^60^ and (2) reproducing the RF model on the GEE platform^50^ by applying the same hyperparameters for generating predictions. This workflow was designed to take advantage of both the efficiency of fine-tuning hyperparameters on the local machine (hyperparameter fine-tuning on GEE is relatively tricker) and the power of cloud computation for model prediction, i.e., large scale LCZ mapping. During the local model fine-tuning, we used cross validation to evaluate model performance (see more details in **Methods for model evaluation**). Once we achieved the best local model performance, we applied the same hyperparameters to build a GEE model and generated the preliminary prediction surfaces across CONUS at 100 m spatial resolution. It’s worth noting that the RF parameters by the scikit-learn and GEE package have different names, but some serve the same function (e.g., n_estimators by scikit-learn vs. numberOfTrees by GEE). Important hyperparameters for our final RF model included 50 decision trees, a minimum of 4 samples to create a leaf node, etc. Furthermore, to reduce the “salt-and-pepper” effect (e.g., misclassification of individual pixels into disparate classes despite their similarity to neighboring pixels), we applied post-classification processing to further improve the model results. We adopted the spatial Gaussian filter developed by Demuzere *et al*.^47^ Briefly, this approach applies Gaussian kernels with LCZ class-dependent standard deviation (*σ*_*i*_) and kernel size (>2*σ*_*i*_) to reduce noise in the predictions. Specifically, we chose *σ*_*i*_ values of 100 m for LCZ 1, 250 m for LCZs 8 and 10, 150 m for the rest of urban classes; we chose 150 m for LCZ E, 25 m for LCZ G and 100 m for other natural classes^47^. As mentioned in Demuzere *et al*.^47^, the choice of *σ*_*i*_ was derived by the priori knowledge from experts. For example, LCZ 1 zones across global cities are typically confined to rather small areas in a city. LCZs 8 and 10 are typically rather large zones. For other urban classes, 150 m is chosen as a scale that is slightly larger than the pixel resolution to ensure that single LCZ pixels do not constitute an LCZ class. For natural classes, the scales are smaller to make sure it is still possible to retain (often small scale) natural features within an urban environment, such as a river/canal, or a small urban park or grass field. Such prior knowledge deserves further investigation and adjustment in future studies and optimal *σ*_*i*_ are assumed to differ among regions^35,47^. After the Gaussian filter, we applied a temporal filter to improve temporal consistency among predictions. Specifically, for each pixel in our LCZ maps, we used a 5-year window (target year ± 2 year) to smooth any abrupt temporal change. The mode of the 5-year window was chosen as the result of the temporal majority vote. When multiple mode values were found, the original classification was kept for these edge cases.

### Methods for model evaluation

We conducted 5-fold spatial cross validation for model evaluation in an effort to mitigate the impact of spatial autocorrelation. Spatial autocorrelation can inflate model test accuracy yet can lead to failure in model generalization and final LCZ mapping^61^. Previous studies have offered approaches to address this issue and reduce the impact of spatial autocorrelation in model performance evaluation by setting a minimum threshold for distance between two training samples, using polygon hold-out or city hold-out strategies to split training and testing sets^55,56,62^. In our study, we created 1000 m × 1000 m grids across CONUS with a unique geohash code for each spatial grid (detailed results of sensitivity analysis by grid size can be found in Supplementary Information: **Sensitivity analysis for spatial hold-out strategy** and Table S3). The LCZ training samples were then linked to the corresponding grid cells. We randomly divided the LCZ training samples into 5 groups based on the spatial geohash code and conducted 5-fold cross validation. This spatial hold-out method ensured that no samples collected from the same 1000 m spatial grid and TA polygon can be both in the train and test sets. Since LCZ TAs are 500 m × 500 m and we only sampled from the inner 200 m × 200 m areas of TAs, our spatial hold-out train-test splitting strategy is stricter than or at least as strict as the polygon hold-out method.

We reported several metrics for model comparison based on the 5-fold spatial cross validation, including the overall accuracy (OA), overall accuracy for urban classes (OA_u_), overall accuracy for built versus natural classes (OA_bu_), weighted accuracy (OA_w_), and class-wise F1 score. The F1 score is the harmonic mean of precision (i.e., the proportion of positive predictions that are actually correct) and recall (i.e., the proportion of actual positives that are correctly identified). The weighted accuracy was designed to account for the (dis)similarity between LCZ classes, such that misclassification between dissimilar LCZ classes (e.g., an urban class and a natural class) received more penalty than misclassification among similar classes (e.g., a midrise class and a lowrise class)^63,64^. To check temporal consistency, we reported normalized average transition rate for each LCZ category. Specifically, we calculated the number of pixels for each LCZ class in each year and created a confusion matrix for every two consecutive years across the modelled 35 years. We then averaged the confusion matrices to obtain yearly transition rates. For straightforward comparison, we normalize the yearly confusion matrix by the LCZ category.

In addition to traditional accuracy assessment, we also conducted a thematic benchmark against an external dataset, i.e., the National Land Cover Database [NLCD]^65^. The NLCD 2019 dataset provides both land cover classification and impervious surface information for 8 years during 2001 and 2019. Considering the various criteria for different classification schemes, we chose to conduct the thematic benchmark based on the impervious fraction. Stewart and Oke provided the range of impervious surface fraction for each LCZ category^25^. According to their values, all natural LCZ classes except LCZ E (bare rock or paved) have impervious surface fraction less than 10%, while all urban LCZ classes except LCZ 9 (sparsely built) are above 10%. In addition, the range of impervious surface fraction for many urban LCZ classes overlap with each other, making it difficult to directly compare impervious surface fraction. Hence, we converted both the NLCD and LCZ datasets into binary maps with a threshold of 10% impervious percent, considering pixels with more than 10% impervious as urban and the other as natural. For our LCZ dataset, since it is possible for LCZ 9 (sparsely built) to be either above or below the threshold, we removed the LCZ 9 pixels during thematic benchmarking. Assuming that the NLCD dataset reports the ground truth, we compared the two binary datasets for 8 years and report the overall accuracy (OA) and the F1 score for each class. We conducted the comparison not only for CONUS, but also for the urbanized areas across CONUS (abbreviated as CONUS-UA) based on the Census 2020 Urban Areas boundaries, since the urban areas are more of interest for the urban studies. Additionally, we compared against an existing CONUS-wide LCZ product that is available for 2017 by Demuzere *et al*.^47^ Since this product doesn’t include LCZ 7 and LCZ 9, we excluded pixels that are classified as LCZ 9 in our LCZ map from the corresponding year. Metrics including OA, OA_u_, OA_bu_, and OA_w_ were calculated and reported.

## Data Records

Our CONUS time-series LCZ maps provide LCZ classification across the continental US spanning 35 consecutive years (1986–2020) with high spatial resolution (100 m). The projection of the LCZ product is USA Contiguous Albers Equal Area Conic (EPSG = 5070). The annual LCZ maps are delivered in the Geo TIFF file format and presented individually for each year. In addition to the final LCZ product, this study also provides LCZ maps of raw results without post-classification to allow data users the flexibility to employ different post-classification techniques as needed. The Training Areas are provided in the form of shapefiles. All LCZ maps and TAs are available via the figshare platform^66^. All the model input feature layers are publicly accessible. The Landsat and LCMS input layers are freely available through Google Earth Engine (https://earthengine.google.com/). The LCMAP layers are freely available through U.S. Geological Survey’s Earth Resources Observation and Science (EROS) Center (https://www.usgs.gov/special-topics/lcmap). The Census data are freely available through the IPUMS National Historical Geographic Information System (NHGIS at https://data2.nhgis.org/main) and the Longitudinal Employer-Household Dynamics (LEHD at https://lehd.ces.census.gov/data/#lodes).

## Technical Validation

### Model accuracy assessment

**Fig. 3:**
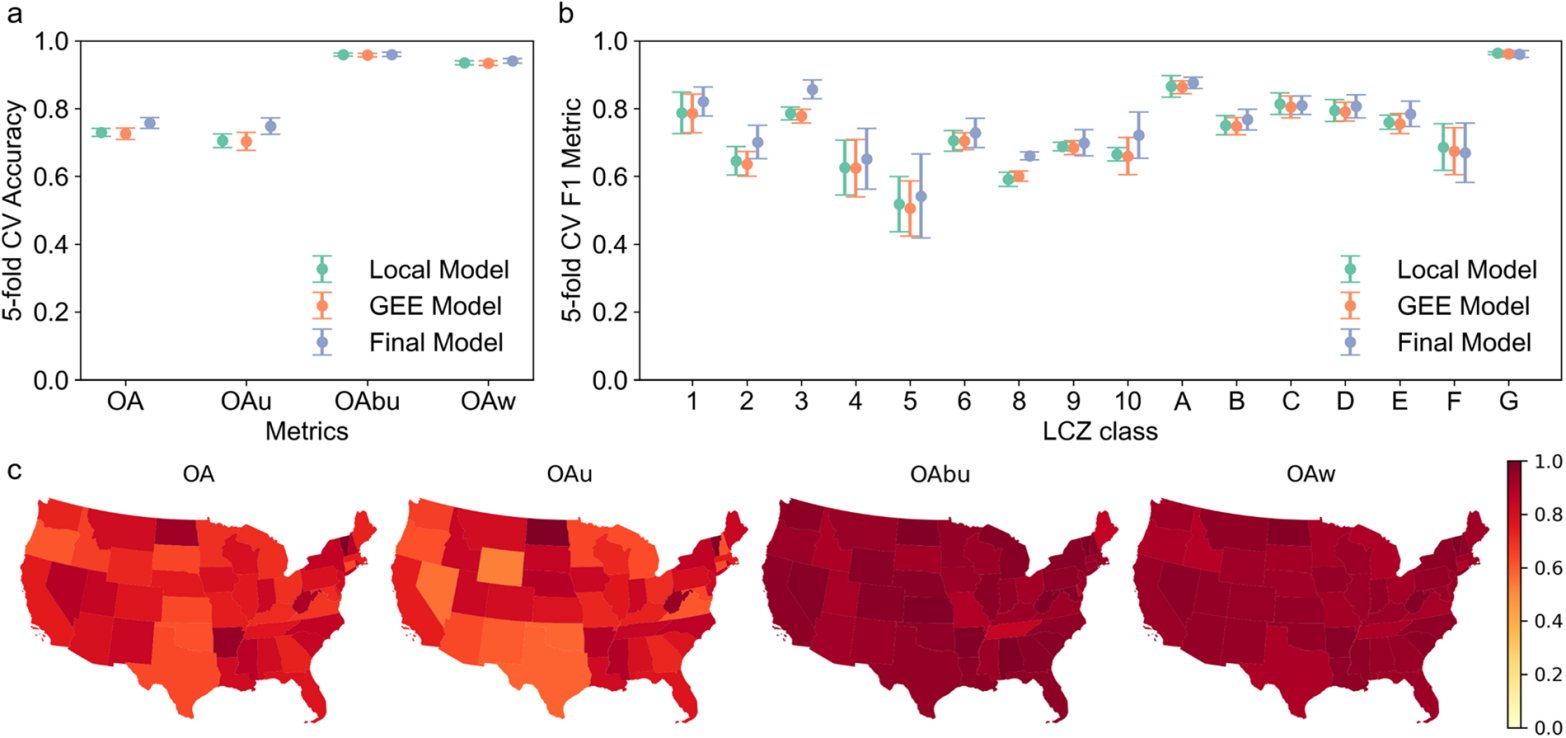
Model performance based on the 5-fold spatial cross validation. (**a**) Overall model performance by the local model, GEE model, and final model (i.e., GEE model with spatial-temporal post-classification processing). Reported metrics include the overall accuracy (OA), overall accuracy for urban classes (OA_u_), overall accuracy for built versus natural classes (OA_bu_), and weighted accuracy (OA_w_). (**b**) Class-wise model performance by the local model, GEE model, and final model. (**c**) Spatial distribution of the final model performance by state. Reported metrics include OA, OA_u_, OA_bu_, and OA_w_.

We developed different LCZ classifiers as part of our hybrid modeling pipeline: a local model, a GEE model, and our final model (i.e., the GEE model combined with spatial-temporal post-classification processing). Figure 3a shows the overall model performance at different stages of our modeling pipeline. As expected, the local and GEE models had very similar performance. The application of the spatial-temporal post-classification processing further increased the model performance by 0.03 for OA and 0.05 for OA_u_. In summary, our final LCZ model achieved 0.76 for the overall accuracy (OA), 0.75 for the overall accuracy of the urban classes (OA_u_), 0.96 for the overall accuracy of the built versus natural classes (OA_bu_), and 0.94 for the weighted accuracy (OA_w_).

To illustrate the spatial heterogeneity in model performance, we compared OA, OA_u_, OA_bu_, and OA_w_ by state using our final LCZ model (Fig. 3c). The state-level OA varies from 0.61 to 0.99, with the lower OA generally occurring in central CONUS. State-level OA_u_ shows the largest variation among the four overall accuracy metrics, ranging from 0.53 to 1.00. For OA_bu_ and OA_w_, all states show high model performance (i.e., 0.85–1.00 for OA_bu_, 0.89–1.00 for OA_w_). To investigate the impact of the spatial distribution of TAs on model performance, we summarized the counts of TAs for each state (Fig. S2). The distribution of TAs is highly right skewed. States such as California, Texas and Illinois have more TAs than other states. However, the number of TAs doesn’t show significant relationship with OA, OA_u_, OA_bu_, or OA_w_ (Fig. S2c,d), indicating the robustness of our model.

**Fig. 4:**
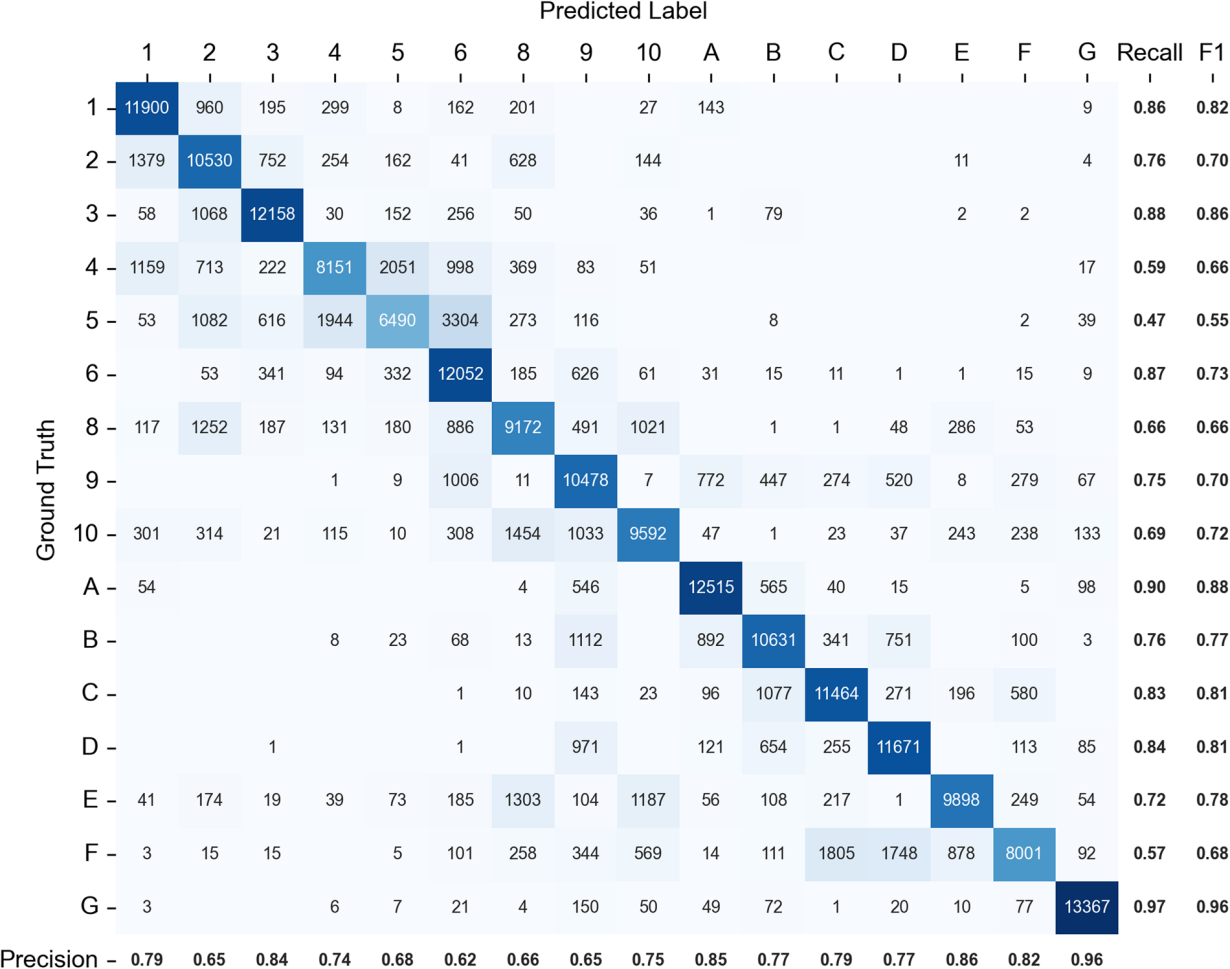
Confusion matrix for the final LCZ model. The value in each cell is the number of test samples. Precision is shown to the bottom of the confusion matrix. Recall and F1 scores are shown to the right of the confusion matrix.

Our final LCZ classifier showed a range of performance by LCZ class (Fig. 3b). Natural LCZ classes (5-fold CV F1 metric: 0.68–0.96) generally had better performance than urban classes (F1: 0.55–0.86). The confusion matrix for all LCZ classes is shown in Fig. 4. LCZ 3 (compact lowrise; F1: 0.86) and LCZ 1 (compact highrise; F1: 0.82) achieved the best performance among the urban classes. LCZ 5 (open midrise; F1: 0.55) and LCZ 4 (open highrise; F1: 0.66) performed worst, which is an issue also identified in other studies^47,48,67^. A potential reason is that many urban classes (e.g., LCZs 4 and 5) share similar surface cover fractions with their main difference being building height. Our model inputs lack features that can distinguish among building heights which may result in misclassification to similar LCZ types. For example, LCZ 5 (open midrise) was misclassified mostly as LCZ 6 (open lowrise) and LCZ 4 (open highrise); LCZ 4 (open highrise) was misclassified mostly as LCZ 5 (open midrise) (Fig. 4). This misclassification may or may not be significant depending on the application, however in general, misclassifying between two similar classes (e.g., compact highrise and compact midrise) is considered less problematic than misclassifying between dissimilar categories (e.g., compact highrise and heavy industry). For the natural classes, LCZ G (water) and LCZ A (dense trees) performed best with F1 scores of 0.96 and 0.88, respectively. LCZ F (bare soil or sand) showed relatively bad performance and was misclassified mainly as LCZ C (bush, scrub) and LCZ D (low plants). One explanation for this result may be the phenological change where certain areas may have varying LCZ classes by season (e.g., dry and wet seasons). Our model misclassification is primarily among similar class types (e.g., LCZ 5 misclassified as LCZs 6 and 4; LCZ 4 misclassified as LCZ 5; LCZ F misclassified as LCZs C and D). These misclassifications generally have high weighted accuracy^63,64^ (i.e., larger than 0.8) thus are less problematic.

**Fig. 5:**
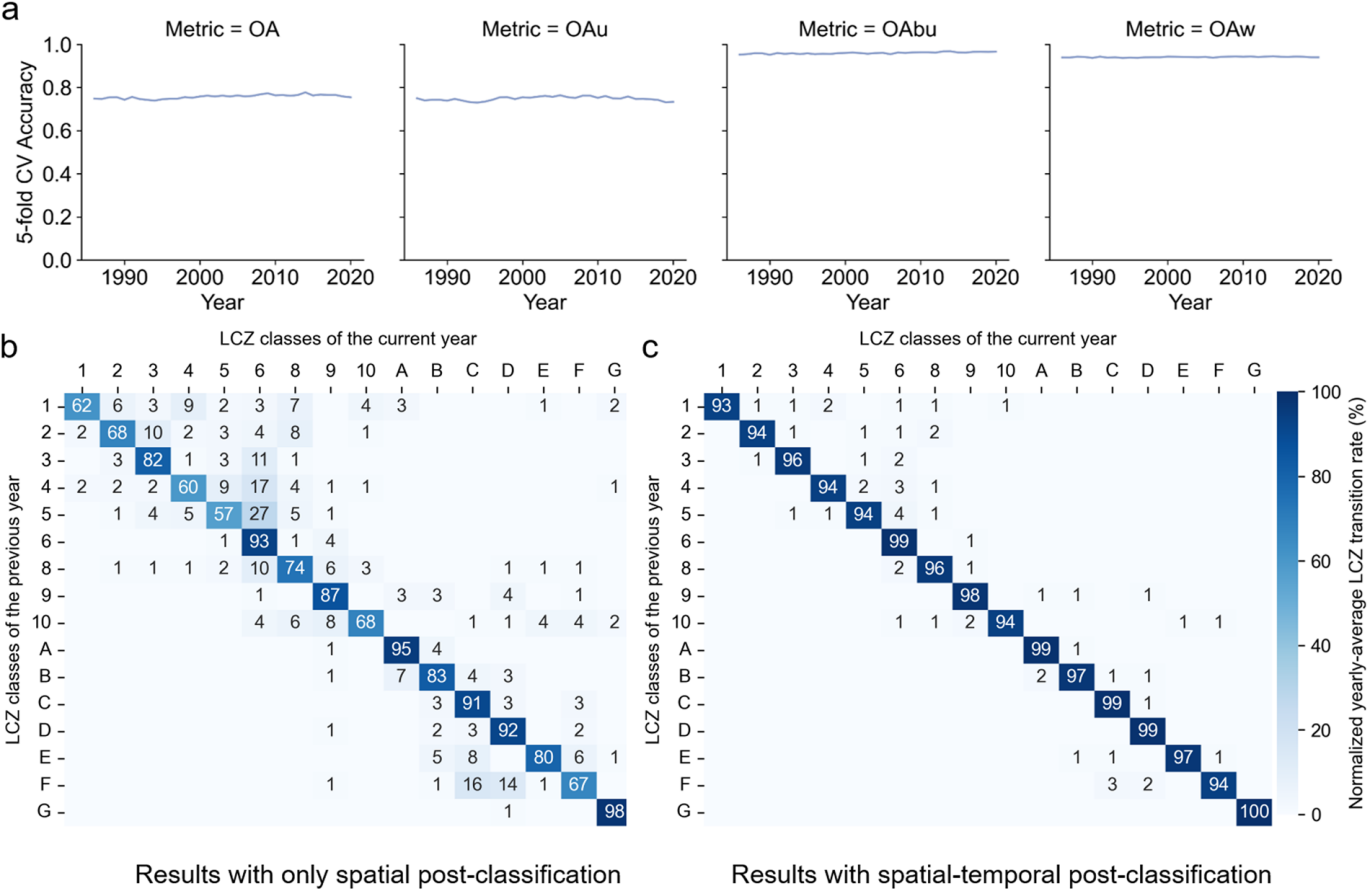
Temporal consistency for the final LCZ model. (**a**) Overall model performance from 1986 to 2020, including the overall accuracy (OA), overall accuracy for urban classes (OA_u_), overall accuracy for built versus natural classes (OA_bu_), and weighted accuracy (OA_w_). (**b,c**) show the average yearly transition (unit: %) among LCZ categories from 1986 to 2020 for CONUS. (**b**) shows the transition for the GEE model with only spatial filtering. (**c**) shows the transition of our final LCZ model, i.e., the GEE model with both spatial and temporal filtering. The transition direction is from rows to columns. For example, in (**c**) row 1, 93% of LCZ 1 pixels in CONUS remained LCZ 1 from a previous year to the current year on average.

We also examined model performance by year (Fig. 5). Overall accuracy metrics were consistent over time. The WUDAPT LCZ classifier includes a spatial post-classification step. Since our objective is to create time-series LCZ maps, we introduced a temporal filter to reduce abrupt LCZ changes in consecutive years for any single pixel. Compared to the LCZ model that only applied a Gaussian spatial filter, adding a temporal filter further increased OA and OA_u_ by 0.01. More importantly, the temporal consistency of our time-series LCZ maps significantly improved. As shown in Fig. 5b,c, the average yearly LCZ transition became more consistent after temporal smoothing. This consistency is expected since land use types change slowly. Some model misclassification persists, e.g., Fig. 5c shows 1% of LCZ 1 pixels across the CONUS converted to LCZ 2 from a previous year to the current year on average, which is unlikely and may be from model misclassification.

In addition to model accuracy assessment, we compared the feature importance scores among different model input features. As shown in Fig. S3, the Landsat features contributed most to our LCZ model. The sum of the feature importance scores for Landsat, LCMS, LCMAP, and Census predictors are 0.54, 0.18, 0.17, and 0.09, respectively. In terms of the individual contribution from a model predictor, population density, BCI, and Landsat shortwave infrared 1 surface reflectance were the three most important features. Compared to solely relying on Landsat imagery, our modeling approach that incorporates LULC time-series features is able to improve the model temporal consistency since the LULC layers are usually derived from several change-detection algorithms^58^. Constrained by the data availability throughout our entire study period, very limited auxiliary data (i.e., LCMAP, LCMS and Census data in this work) is available for model development and improvement. The lack of suitable features that include information on building heights and other urban structure characteristics is one of the reasons for confusion between some urban categories. The inclusion of total population partially mitigated this issue since the distribution of population is closely related to density of buildings. Our attempt to include segmented employment data into the model also showed further improvement in accuracies over urban classes. However, it’s worth noting that the total population is not available for each year and interpolation was used for non-decennial years, which can be one factor of model uncertainty.

### Longitudinal LCZ maps for CONUS

**Fig. 6:**
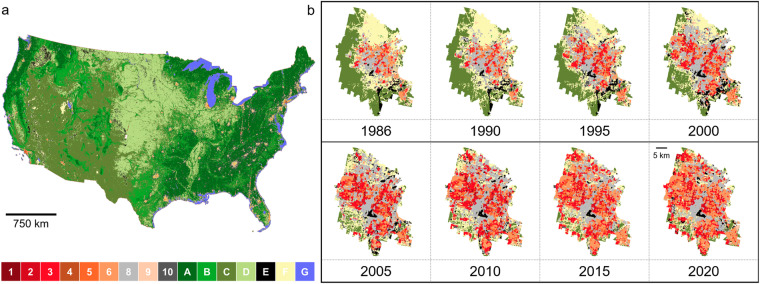
LCZ mapping. (**a**) CONUS prediction surface at 100 m × 100 m resolution using year 2020 for illustration. (**b**) LCZ mapping for an example city (Las Vegas, Nevada) at 100 m × 100 m resolution by year. For illustration, change in urban form is shown based on a ~5-year interval and the Census 2020 Urban Areas boundary. Full results for all years (1986 to 2020) are available in our LCZ dataset.

We applied our final LCZ model to make prediction surfaces with high spatial resolution (i.e., 100 m × 100 m) for CONUS from 1986 to 2020. Figure 6a shows the LCZ map for 2020 for illustration. The spatial pattern of our LCZ maps is generally consistent with a previously published CONUS LCZ map representative for the year 2017 with an overall agreement of 0.73^47^. Specifically, we achieved 0.73 for OA, 0.87 for OA_u_, 0.99 for OA_bu_, and 0.95 for OA_w_ when compared to this single-year LCZ map (see **Methods for model evaluation**). To our knowledge, this study is the first that develops a LCZ dataset which spans 35 years across CONUS. Our dataset may enable various longitudinal analyses (i.e., from 1986–2020) including urban sprawl analyses, urban heat analyses, urban climate and weather modeling, etc. For example, Las Vegas is well-known as one of the fastest growing cities in the US. The LCZ maps extracted from our dataset clearly show the expected urban expansion of Las Vegas (Fig. 6b). For illustration, we used the 2020 Las Vegas urbanized area boundary to calculate the proportion of urban pixels within the boundary for each year. In 1986, only 29% of the pixels were classified as urban LCZ classes; in 2020 the proportion increased to 81%. The proportion of compact classes (i.e., LCZs 1–3) increased from 3% in 1986 to 21% in 2020 and from 11% to 36% for open urban classes (i.e., LCZs 4–6).

**Fig. 7:**
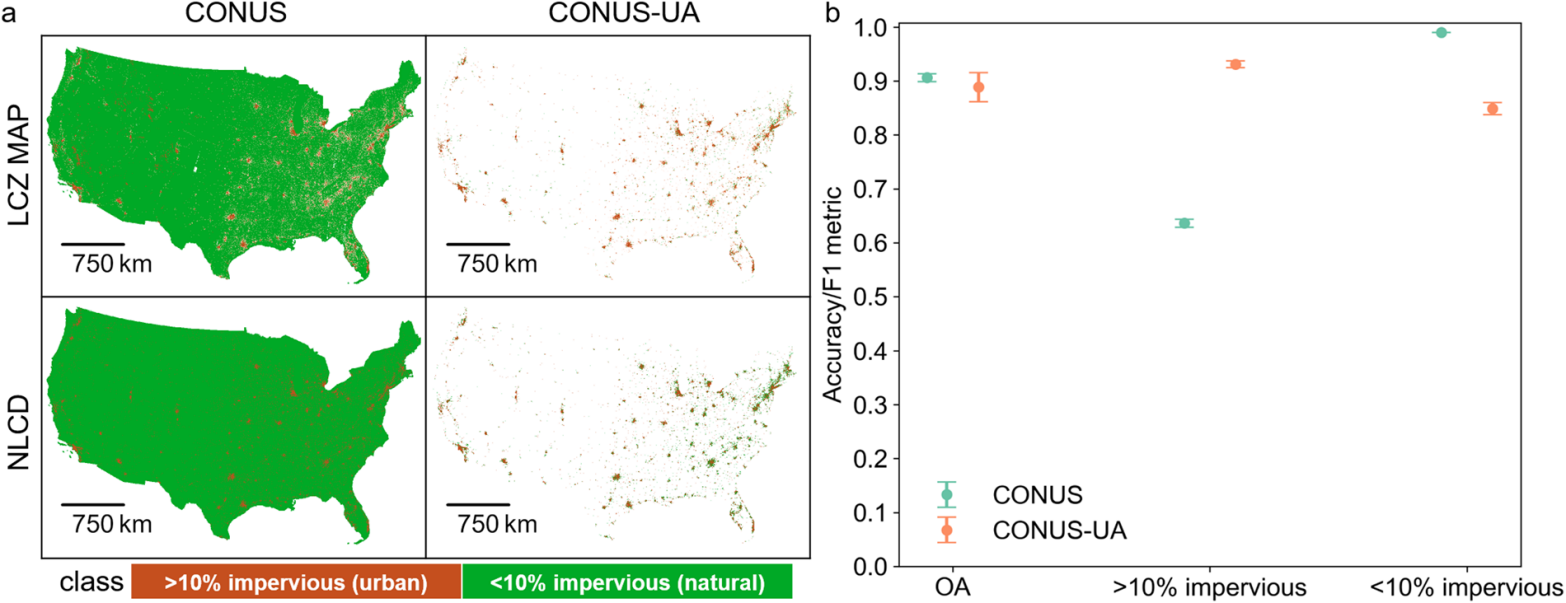
Thematic benchmarking. (**a**) The binary (urban vs. natural) LCZ and NLCD maps using 2019 for illustration. The threshold for defining urban vs. natural was 10% impervious surface fraction. LCZ 9 (sparsely built) was not considered in the thematic benchmarking because the impervious fraction of LCZ 9 pixels may fall into either category. The comparison was conducted for both CONUS, and CONUS-UA which used Census 2020 Urban Areas boundary. (**b**) Comparison results for overall accuracy and the urban and natural pixels.

We conducted thematic benchmarking using the National Land Cover Database for 8 available years based on the impervious fraction. We created binary variables for both the LCZ and the NLCD maps into two classes (urban vs. natural) using a threshold of 10% impervious surface fraction. Since there are far more natural pixels than urban pixels across the entire CONUS, we compared the two datasets not only for CONUS but also for only the urban areas within CONUS [CONUS-UA]. As shown in Fig. 7b, the overall consistency between the two datasets was satisfactory, with average OAs of 0.91 and 0.89 for CONUS and CONUS-UA, respectively. The class-wise F1 scores across CONUS were 0.63 for the urban pixels (i.e., > 10% impervious fraction) and 0.99 for the natural pixels (i.e., < 10% impervious fraction). The relatively lower performance of the urban class was attributed to the discrepancies between the NLCD and LCZ datasets in identifying some urban pixels, especially in regions outside the CONUS-UA. However, within CONUS-UA, both urban and natural classes performed well, with F1 scores of 0.93 and 0.85, respectively. These results indicate that our LCZ maps achieved satisfactory and robust consistency to an independent, high-quality national land cover dataset.

### LCZ distribution and change mapping

**Fig. 8:**
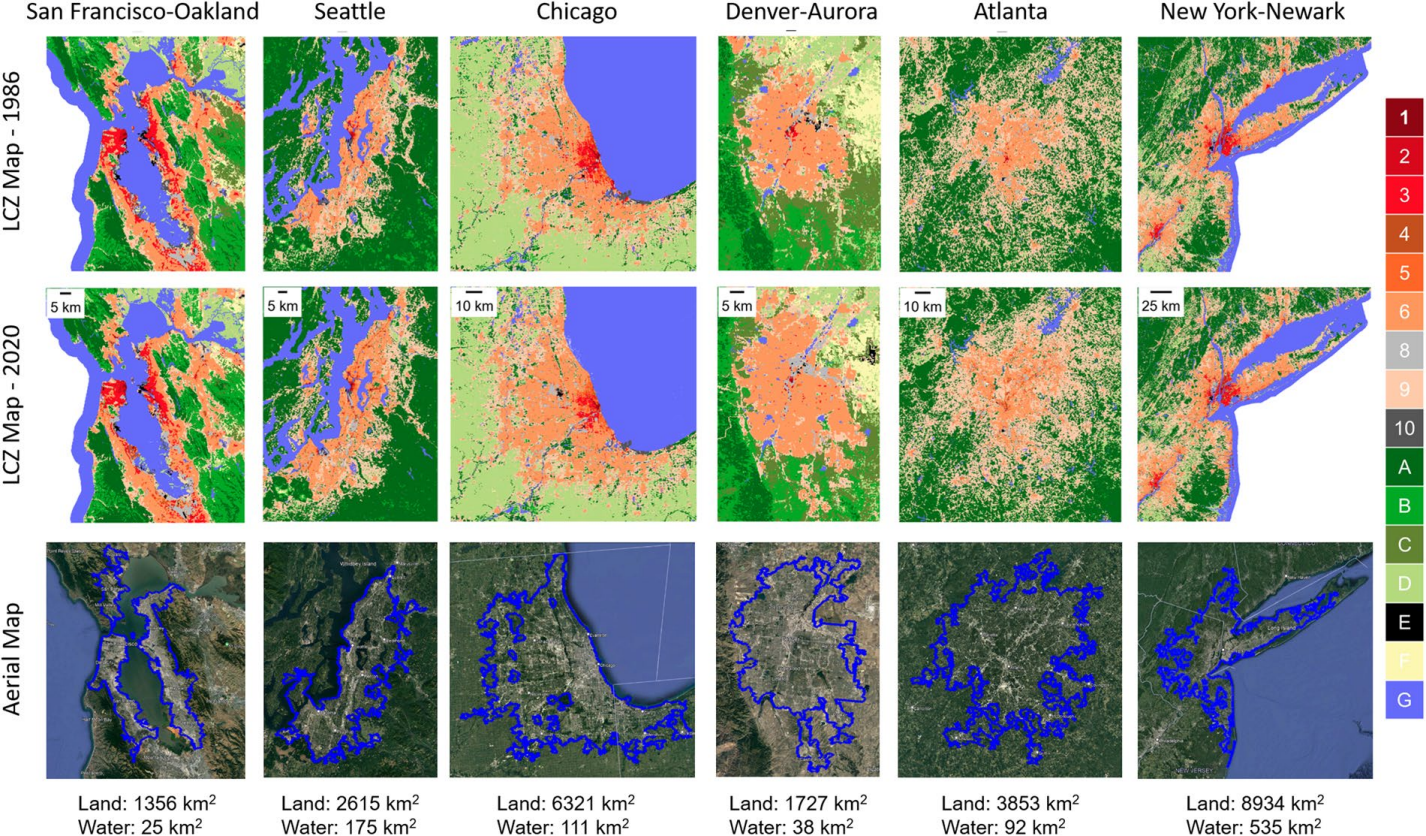
LCZ maps for 6 example metropolitan areas (two cities each for the western, central, and eastern US) in 1986 and 2020 with corresponding aerial maps from Google Earth (© Google Earth 2020). Blue lines in the aerial maps are the Census 2020 Urban Areas boundaries. Land and water area within the boundaries are listed for reference.

To further validate our product, we investigated additional metropolitan areas in the US and mapped their LCZ distributions and land use change over time. Specifically, we used 6 metropolitan areas in the US (i.e., San Francisco-Oakland, Seattle, Chicago, Denver-Aurora, Atlanta, and New York-Newark) to summarize the LCZ distribution and change over 35 years. We calculated the proportion of pixels for each LCZ category within the boundary of each metropolitan area. The transition among LCZ categories were tracked between 1986 and 2020. We calculated the absolute number of pixels that were LCZ X in 1986 and turned to LCZ Y in 2020 (e.g., pixels that were LCZ 9 in 1986 and converted to LCZ 6 in 2020). We then divided by the total number of LCZ pixels in 1986 for normalization. For consistency, all calculations were based on pixels within the Census 2020 Urban Areas boundaries. Figure 8 shows the LCZ maps of the example cities using 1986 and 2020 for illustration. The aerial maps show the Census 2020 Urban Areas boundaries. The land and water area are listed alongside for context when comparing among cities. By comparing the LCZ distribution pattern between 1986 and 2020, Denver-Aurora and Atlanta show the most obvious urban sprawl patterns.

**Fig. 9:**
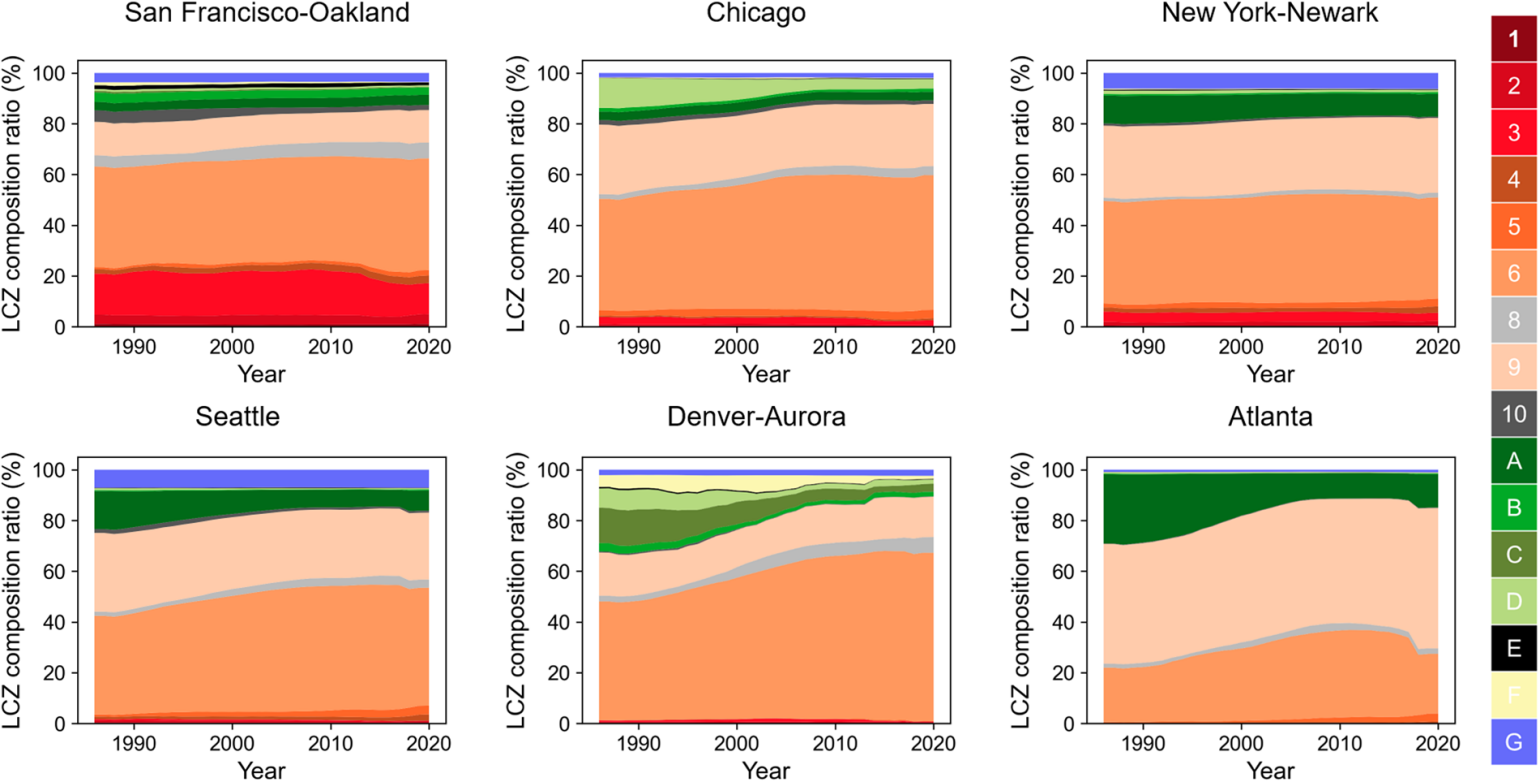
Trends of LCZ composition ratio from 1986 to 2020 for 6 US metropolitan areas. All composition ratios were calculated based on the Census 2020 Urban Areas boundary shown in Fig. 8.

**Fig. 10:**
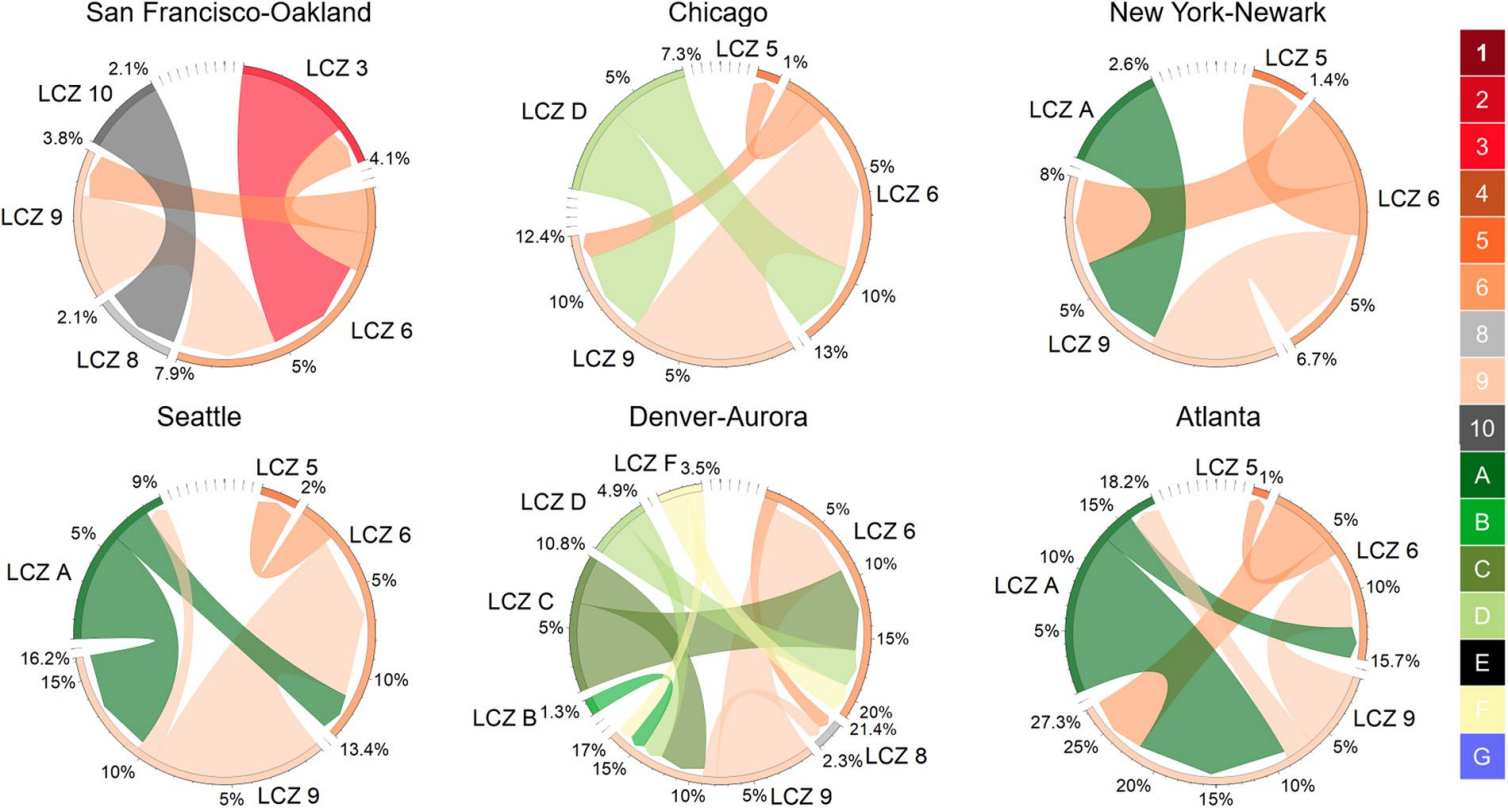
The chord diagram to show the major transition of LCZ classes between year 1986 and year 2020 for 6 US metropolitan areas. The directions of the transitions are denoted by the arrows. The colors of the chord are consistent with the colors of the transition sources. The ticks on the arcs represent the transition rate. For example, as shown by the LCZ C arc in Denvor-Aurora, 7% of pixels transit from LCZ C to LCZ 6 (chord color consistent with LCZ C); 4% from LCZ C to LCZ F (chord color consistent with LCZ C); 0% from other LCZ types to LCZ C. To illustrate major transitions, any LCZ transition that occurred in less than 1% of city pixels were removed from the chord diagram; pixels remaining the same type were also removed. Full transition details can be found in Supplementary Information Fig. S4. All transition rates were calculated based on the Census 2020 Urban Areas boundary shown in Fig. 8.

As shown in Fig. 9, 10, our longitudinal LCZ dataset is capable of measuring change over time of LCZ composition and tracking where major transitions occurred. Among the 6 example cities, the fastest urban expansion (i.e., large increases in urban LCZ ratios) occurred in Denver-Aurora. In 1986, natural LCZ classes covered 32% of the Denver-Aurora area – mostly LCZ C (bush, scrub), LCZ D (low plants), and LCZ F (bare soil or sand). During urban expansion across 35 years, the proportion of natural classes continuously shrunk, and the composition ratio of the natural classes decreased to 11% in 2020 (Fig. 9). The LCZ type that grew the most was LCZ 6 (open lowrise) with a 20% increase in composition ratio – 8% was converted from pixels of LCZ 9 (sparsely built), 7% from LCZ C (bush, scrub), 3% from LCZ D (low plants) and 2% from LCZ F (bare soil or sand) (Fig. 10). Similarly, Atlanta, Chicago, and Seattle also showed signs of urban sprawl. Like Denver-Aurora, both Chicago and Seattle had the most growth in LCZ 6 (open lowrise) with an increase of 9% and 8% in area percentage, respectively. The major transition was both from LCZ 9 (sparsely built) to LCZ 6 (open lowrise). In Atlanta, the major source of urban sprawl came from deforestation and forest fragmentation^68^. In 1986, LCZ A (dense trees) were 27% of the city area compared to only 13% in 2020. Most of the dense trees lost were converted to LCZ 9 (sparsely built). Another main transition was that 6% of pixels were converted from LCZ 9 (sparsely built) to LCZ 6 (open lowrise) demonstrating the gradual urban development and expansion in Atlanta. We also observed an abrupt drop in the composition ratio for LCZ 6 (open lowrise) in 2018. This might be a result of misclassification from LCZ 6 (open lowrise) to LCZ 9 (sparsely built). The other two example cities (San Francisco-Oakland and New York-Newark) showed relatively slower urban expansion during the study period. This could be partially explained by major urban expansion in these two urban agglomerations before 1986. Still, we found 3% pixels converted from LCZ 9 (sparsely built) to LCZ 6 (open lowrise) and 2% pixels converted from LCZ 10 (heavy industry) to LCZ 8 (large lowrise) in San Francisco-Oakland from 1986 to 2020. In New York-Newark, 3% pixels converted from LCZ 9 (sparsely built) to LCZ 6 (open lowrise) and 3% from LCZ A (dense trees) to LCZ 9 (sparsely built).

Figure. S5 shows more localized LCZ mapping using the New York County and San Francisco County for illustration. Satellite imagery for sample TAs in these two regions are also shown to demonstrate the prediction results versus the real labels. Overall, our crowdsourced-expert hybrid labeling was scalable and efficient. The local-cloud hybrid modeling approach leveraged the speed of fine-tuning model hyperparameters on a local machine while leveraging the power of model transfer and reproduction on a cloud platform (GEE) for making predictions at a large scale. Our lightweight LCZ classifier achieved good model performance (e.g., 0.76 for overall accuracy and 0.94 for weighted accuracy) and showed good consistency with NLCD (an external high-quality land cover dataset) for measuring the footprint of built-up areas. In summary, our modeling framework could be applied to other areas for large-scale longitudinal LCZ mapping. Our LCZ dataset has potential to support a wide range of research fields, such as urban weather and climate modeling, urban expansion and development, risk analysis of various urban hazards, etc. Moreover, our longitudinal LCZ dataset can serve as a valuable resource for urban planners, policy makers, and researchers to assess local, regional, and national policies related to urbanization, supporting informed decision-making for sustainable urban development.

### Supplementary information

Supplementary Information for “Mapping urban form into local climate zones for the continental US from 1986-2020”

## Data Availability

Python scripts for training data sampling, earth observation and census input feature collection, random forest model fine tuning and mode prediction on Google Earth Engine are available at https://github.com/QiMengEnv/CONUS_Longitudinal_LCZ. All data processing and visualizations are done in Python 3.9. The post-classification processing is done in JavaScript on Google Earth Engine Code Editor and is also available with the same URL.
