# Supplementary material for: Mapping urban form into local climate zones for the continental US from 1986–2020
Source: Sci Data. 2024 Feb 13;11:195. doi: 10.1038/s41597-024-03042-4 (PMC10864375; doi:10.1038/s41597-024-03042-4)
Supplement: Supplementary file 1 — Supplementary Information for “Mapping urban form into local climate zones for the continental US from 1986-2020” [file 41597_2024_3042_MOESM1_ESM.pdf]

Supplementary Information for "Mapping urban form into local climate zones for the continental US from 1986-2020"

Authors
Meng Qi[1], Chunxue Xu[2], Wenwen Zhang[3], Matthias Demuzere[4], Perry Hystad[5], Tianjun Lu[6], Peter James[7,8], Benjamin Bechtel[4], Steve Hankey[1,*]

Affiliations
1. School of Public and International Affairs, Virginia Polytechnic Institute and State University, Blacksburg, VA 24060, United States
2. College of Earth, Ocean, and Atmospheric Sciences, Oregon State University, Corvallis, OR 97331, United States
3. Edward J. Bloustein School of Planning and Public Policy, Rutgers University, New Jersey, 08901, United States
4. Urban Climatology Group, Department of Geography, Ruhr-University Bochum, Bochum, 44801, Germany
5. College of Public Health and Human Sciences, Oregon State University, Corvallis, OR 97331, United States
6. Department of Epidemiology and Environmental Health, University of Kentucky, Lexington, KY 40536, United States
7. Department of Population Medicine, Harvard Medical School and Harvard Pilgrim Health Care Institute, Boston, MA 02215, United States
8. Department of Environmental Health, Harvard T.H. Chan School of Public Health, Boston, MA 02215, United States

corresponding author(s): Steve Hankey (hankey@vt.edu)

Abstract
Urbanization has altered land surface properties driving changes in micro-climates. Urban form influences people's activities, environmental exposures, and health. Developing detailed and unified longitudinal measures of urban form is essential to quantify these relationships. Local Climate Zones [LCZ] are a culturally-neutral urban form classification scheme. To date, longitudinal LCZ maps at large scales (i.e., national, continental, or global) are not available. We developed an approach to map LCZs for the continental US from 1986 to 2020 at 100m spatial resolution. We developed lightweight contextual random forest models using a hybrid model development pipeline that leveraged crowdsourced and expert labeling and cloud-enabled modeling – an approach that could be generalized to other countries and continents. Our model achieved good performance: 0.76 overall accuracy (0.55-0.96 class-wise F1 scores). To our knowledge, this is the first high-resolution, longitudinal LCZ map for the continental US. Our work may be useful for a variety of fields including earth system science, urban planning, and public health. Our hybrid labeling and modeling approach could be generalized to other countries and continents.

Table of Contents

Sensitivity analysis for sampling regions **3**

Table S1. Sensitivity analysis for the impact of sampling regions on model performance based on three scenarios of training and testing set sampling. All the metrics are reported for the local LCZ model. The area parameters represent a square located in the center of the TAs with different side lengths. 3

Table S2. Details of the model input variables.. 4

Incorporating segmented employment data into the LCZ model **5**

Fig. S1. Comparison of model performance (based on the 5-fold spatial cross validation) when incorporating segmented employment data into the LCZ final model. (a) Overall model performance, including the overall accuracy (OA), overall accuracy for urban classes (OA_u), overall accuracy for built versus natural classes (OA_{bu}), and weighted accuracy (OA_w). (b) Class-wise model performance. **5**

Sensitivity analysis for spatial hold-out strategy **6**

Table S3. Sensitivity analysis for the impact of spatial held-out grid size on model performance. All the metrics are reported for the local LCZ model. 6

Fig. S2. The relationship between the distribution of TAs and the model performance. (a) Spatial distribution of TAs by state using 2020 for illustration. We adjusted the color bar scale to visualize the spatial patterns of TAs due to its right skewness and used the 90th percentile as the maximum for visualization. (b) The histogram of TAs summarized by state. (c) Scatter plot of OA vs count of TAs. (d) Scatter plot of OA_u vs count of TAs. (e) Scatter plot of OA_{bu} vs count of TAs. (f) Scatter plot of OA_w vs count of TAs. For (c) to (d), each scatter represents a state. **7**

Fig. S3. Feature importance ranking for the local LCZ model. (a) Feature importance scores aggregated by variable types listed in **Table S2**. (b) Individual feature importance scores for the top 10 important predictors. Landsat variables with different contextual statistics were aggregated. For example, BCI refers to the sum of feature importance scores from BCI_mean, BCI_max, BCI_min, BCI_median, BCI_p25 and BCI_p75. 8

Fig. S4. The chord diagram to show the full details of LCZ transitions between year 1986 and year 2020 for 6 US metropolitan areas. The directions of the transitions are denoted by the arrows. The colors of the chord are consistent with the colors of the transition sources. The ticks on the arcs represent the transition rate. All the transition rates were calculated based on the Census 2020 Urban Areas boundary shown in Fig. 8. .... **9**

Fig. S5. Localized LCZ mapping using New York County and San Francisco County for illustration. Sample TAs were selected for both regions and the corresponding satellite imagery was shown to compare against the real LCZ labels. The year for each LCZ map and satellite imagery was listed below the visualizations. To illustrate the change in LCZ distribution, LCZs in 1995 and 2020 were shown for New York County, and maps in 1993 and 2020 for San Francisco County. The choice of the old year was based on the availability of satellite imagery for each region. The TAs labels were also listed below the visualizations. **10**

References **11**

Sensitivity analysis for sampling regions

As described in section **Hybrid TA labeling**, our LCZ TAs are 500m × 500m digitized polygons for MTurk crowdsourced labeling. While some studies randomly sampled pixels within the entire 500m × 500m TAs[1], our sensitivity analysis using local models indicated that sampling from a smaller area near the center of the TAs can achieve better model performance. Three scenarios were designed for sensitivity analyses. All of them used data sampled from the central area for model training but from different areas for testing set assembly (**Table S1**). As expected, model fits were best when both training and testing were sampled from the central region near the center of the TAs and were slightly worse when the testing set was solely sampled beyond the central area. When using the entire polygon to sample testing set, the model performance was almost the same despite the size of the central area, indicating the authenticity of training labels across 500m × 500m TAs. As shown in **Table S1**, sampling from a 100m × 100m or 200m × 200m area (with the centroid of TAs as the center) for both training and testing achieved the best model performance. We suspect that confining the training set in central area plays a role in reducing the heterogeneity effect within TAs. For example, mixed LCZ types within one polygon isn't uncommon, which is also one of the reasons that inconsistent labels by different urban experts have been reported[2,3]. To allow more training samples, we chose 200m × 200m for model development in this study.

Table S1. Sensitivity analysis for the impact of sampling regions on model performance based on three scenarios of training and testing set sampling. All the metrics are reported for the local LCZ model. The area parameters represent a square located in the center of the TAs with different side lengths.

Scenario	Training	Testing	Metrics	100m × 100m	200m × 200m	300m × 300m	400m × 400m	500m × 500m
1	Central Area	Central Area	OA	0.73	0.73	0.73	0.71	0.69
			OA_u	0.71	0.71	0.7	0.68	0.66
			OA_{bu}	0.96	0.96	0.95	0.95	0.94
			OA_w	0.94	0.94	0.93	0.93	0.92
2	Central Area	Entire Polygon	OA	0.69	0.68	0.68	0.68	/
			OA_u	0.66	0.65	0.65	0.66	/
			OA_{bu}	0.94	0.94	0.94	0.94	/
			OA_w	0.92	0.92	0.92	0.92	/
3	Central Area	Beyond Central Area	OA	0.66	0.65	0.64	0.62	/
			OA_u	0.62	0.62	0.61	0.59	/
			OA_{bu}	0.92	0.92	0.92	0.91	/
			OA_w	0.91	0.90	0.90	0.89	/

Table S2. Details of the model input variables.

Variable Type	Feature Extraction	Variables	Count of Variables
LCZ	Contextual statistics: mean, max, min, median, p25, p75	• Blue,<br>• Green,<br>• Red,<br>• Near-infrared,<br>• Shortwave infrared 1 and 2,<br>• Surface temperature,<br>• The Biophysical Composition Index (BCI),<br>• The mean Normalized Difference BAreness Index (NDBAI),<br>• The minimum and maximum Normalized Difference Vegetation Index (NDVI),<br>• The maximum Normalized Difference Water Index (NDWI)	72
LCMAP	Contextual composition ratio	8 land cover classes in two products, i.e., Primary Land Cover (LCPRI) and Secondary Land Cover (LCSEC):<br>• Developed,<br>• Cropland,<br>• Grass/Shrub,<br>• Tree Cover,<br>• Water,<br>• Wetland,<br>• Ice/Snow,<br>• Barren	16
LCMS	Contextual composition ratio	15 Land Cover types:<br>• Trees,<br>• Tall Shrubs & Trees Mix (SEAK only),<br>• Shrubs & Trees Mix,<br>• Grass/Forb/Herb & Trees Mix,<br>• Barrens & Trees Mix,<br>• Tall Shrubs (SEAK only),<br>• Shrubs,<br>• Grass/Forb/Herb & Shrubs Mix,<br>• Grass/Forb/Herb,<br>• Barren & Grass/Forb/Herb Mix,<br>• Barren or Impervious,<br>• Snow or Ice,<br>• Water,<br>• Non-Processing Area Mask<br>7 Land Use types:<br>• Agriculture,<br>• Developed,<br>• Forest,<br>• Non-Forest Wetland,<br>• Other,<br>• Rangeland or Pasture,<br>• Non-Processing Area Mask	22
Total population	Direct value	• Total population,<br>• Total population density	2
Year	Dummy variable	• Year	1

Incorporating segmented employment data into the LCZ model

Our final LCZ model shows that a few urban classes are hard to classify. As an attempt to further improve the model performance, we incorporated segmented employment data collected from Longitudinal Employer-Household Dynamics - Origin-Destination Employment Statistics dataset [LEHD-LODES] into our LCZ model[4]. This dataset provides employment data from 20 segments, including manufacturing, construction, utilities, wholesale trade, retail trade, etc. Our results showed that adding segmented employment information significantly improved the accuracy for urban classes, especially for LCZ 4 (open highrise) and LCZ 8 (large lowrise), whose F1 score increased 0.08 and 0.07, respectively. The overall accuracy (OA) increased 0.03 while the accuracy for urban classes (OA_u) increased 0.05. However, the segmented employment data was only available from 2002 to 2019, with gaps for several states and cities. This greatly limited its use for our ultimate purpose (i.e., longitudinal LCZ mapping from 1986 to 2020) hence was not included in our final product. Still, this result can be informative for studies whose target study time period falls within 2002 to 2019.

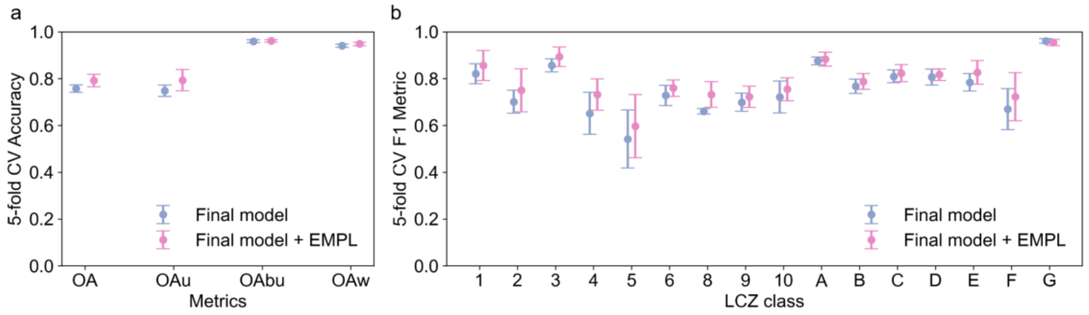

Fig. S1. Comparison of model performance (based on the 5-fold spatial cross validation) when incorporating segmented employment data into the LCZ final model. (a) Overall model performance, including the overall accuracy (OA), overall accuracy for urban classes (OA_u), overall accuracy for built versus natural classes (OA_{bu}), and weighted accuracy (OA_w). (b) Class-wise model performance.

Sensitivity analysis for spatial hold-out strategy

As described in **Methods for model evaluation**, we split training and testing sets based on 1000m × 1000m grids such that no training samples and testing samples were allowed to fall within the same spatial grids. As a sensitivity analysis over the grid size, we reported the impact of grid size on model performance (**Table S3**). As expected, the results showed that the smaller the grid size, the higher the model performance, while when the grid size was small enough, the model was overfit and likely to fail in generalization. In this study, we chose 1000m × 1000m grids as this strategy was already stricter than or at least as strict as the polygon-based hold out strategy[5].

Table S3. Sensitivity analysis for the impact of spatial held-out grid size on model performance. All the metrics are reported for the local LCZ model.

Metrics	100m	500m	1000m	2000m	5000m	10000m
OA	0.86	0.76	0.73	0.72	0.69	0.69
OA_u	0.84	0.74	0.71	0.70	0.66	0.66
OA_{bu}	0.97	0.96	0.96	0.96	0.96	0.95
OA_w	0.96	0.94	0.94	0.93	0.93	0.92

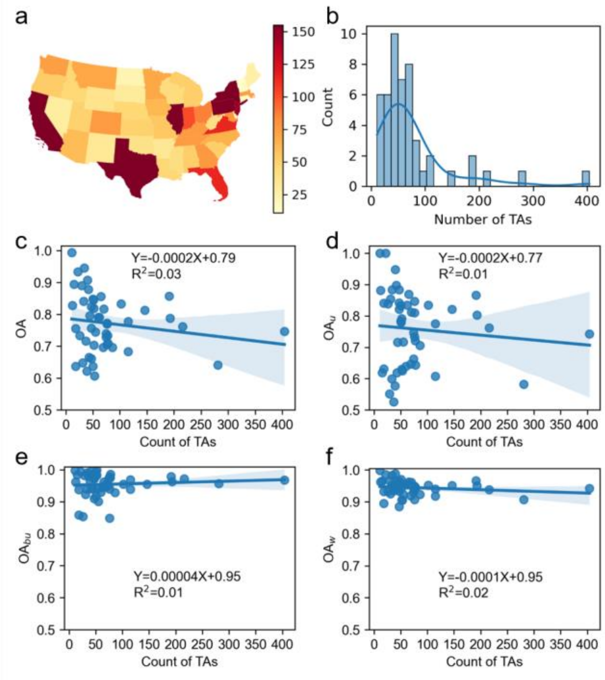

Fig. S2. The relationship between the distribution of TAs and the model performance. (a) Spatial distribution of TAs by state using 2020 for illustration. We adjusted the color bar scale to visualize the spatial patterns of TAs due to its right skewness and used the 90th percentile as the maximum for visualization. (b) The histogram of TAs summarized by state. (c) Scatter plot of OA vs count of TAs. (d) Scatter plot of OA_u vs count of TAs. (e) Scatter plot of OA_{bu} vs count of TAs. (f) Scatter plot of OA_w vs count of TAs. For (c) to (d), each scatter represents a state.

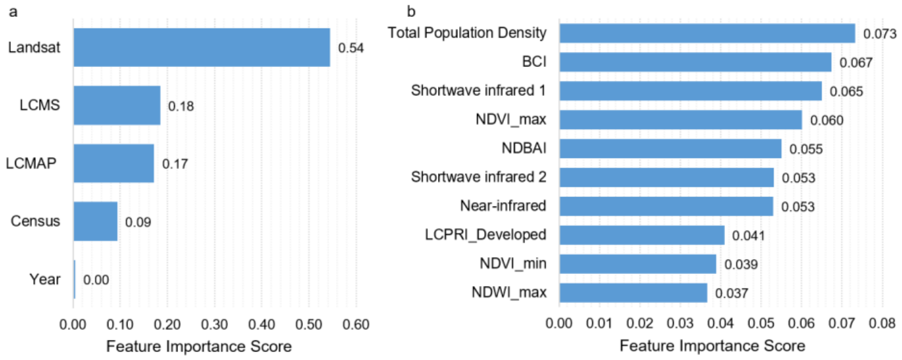

Fig. S3. Feature importance ranking for the local LCZ model. (a) Feature importance scores aggregated by variable types listed in **Table S2**. (b) Individual feature importance scores for the top 10 important predictors. Landsat variables with different contextual statistics were aggregated. For example, BCI refers to the sum of feature importance scores from BCI_mean, BCI_max, BCI_min, BCI_median, BCI_p25 and BCI_p75.

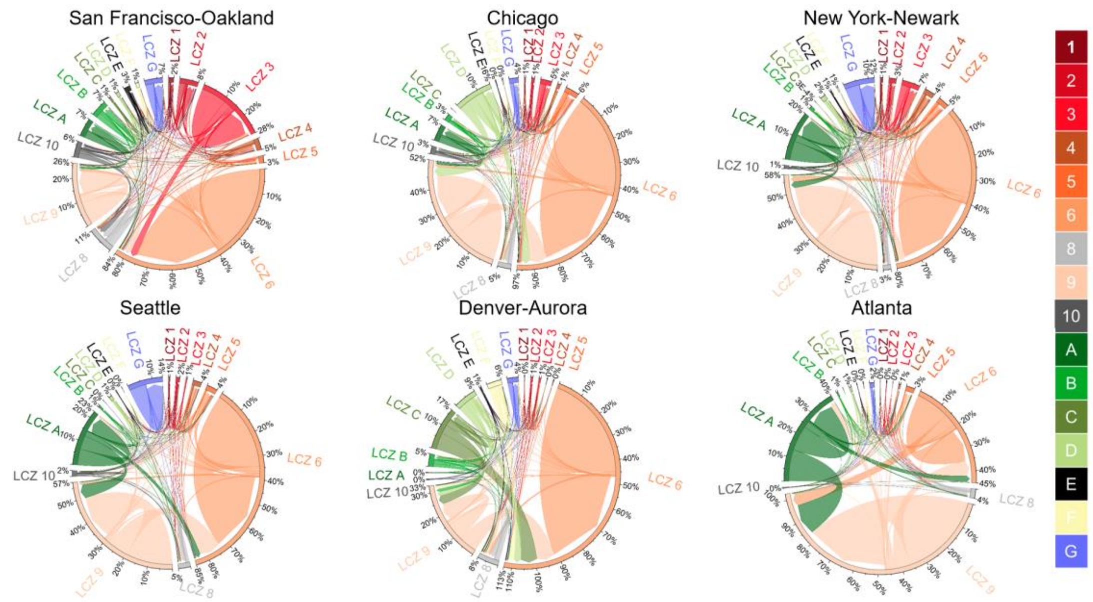

Fig. S4. The chord diagram to show the full details of LCZ transitions between year 1986 and year 2020 for 6 US metropolitan areas. The directions of the transitions are denoted by the arrows. The colors of the chord are consistent with the colors of the transition sources. The ticks on the arcs represent the transition rate. All the transition rates were calculated based on the Census 2020 Urban Areas boundary shown in **Fig. 8**.

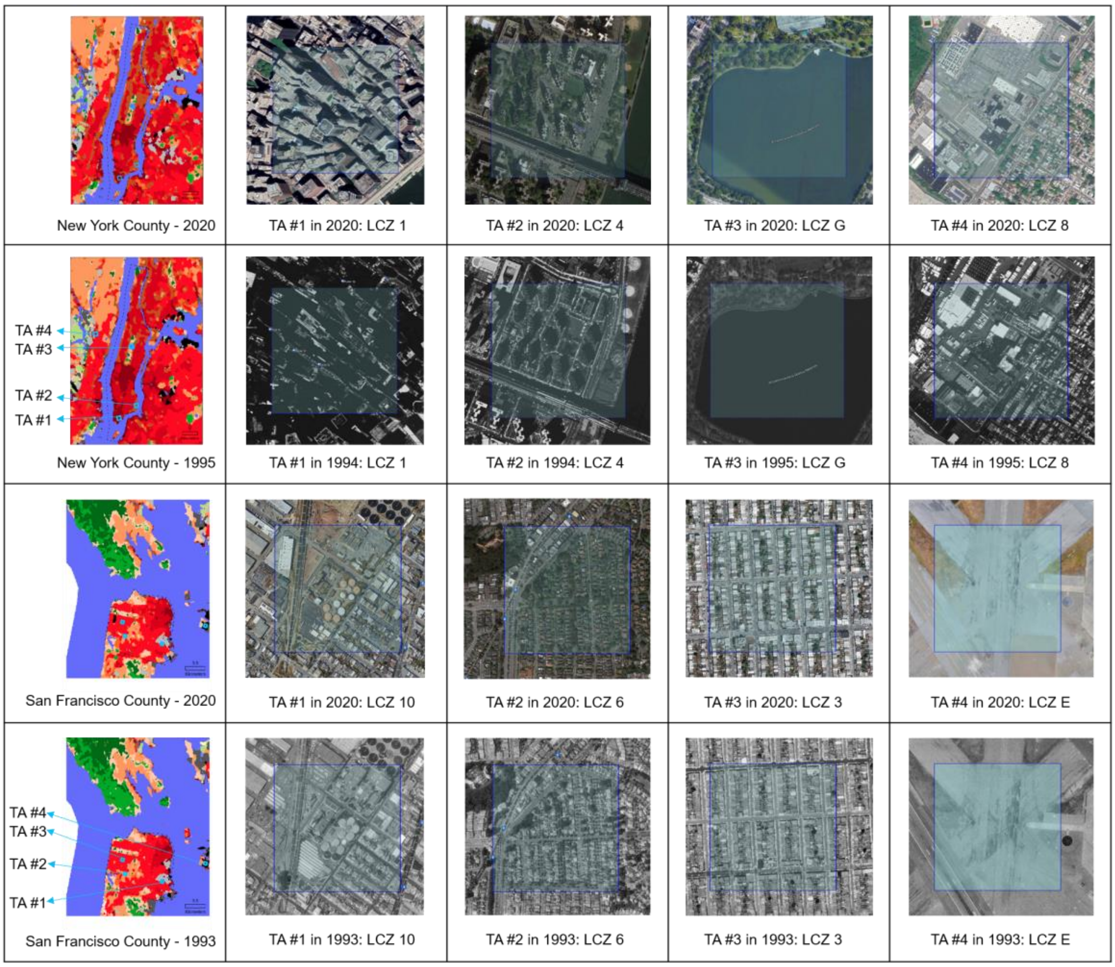

Fig. S5. Localized LCZ mapping using New York County and San Francisco County for illustration. Sample TAs were selected for both regions and the corresponding satellite imagery was shown to compare against the real LCZ labels. The year for each LCZ map and satellite imagery was listed below the visualizations. To illustrate the change in LCZ distribution, LCZs in 1995 and 2020 were shown for New York County, and maps in 1993 and 2020 for San Francisco County. The choice of the old year was based on the availability of satellite imagery for each region. The TAs labels were also listed below the visualizations.

References

1 Demuzere, M. *et al.* Combining expert and crowd-sourced training data to map urban form and functions for the continental US. *Sci. Data* **7**, 1-13 (2020).

2 Xu, C. *et al.* Application of training data affects success in broad-scale local climate zone mapping. *Int. J. Appl. Earth Obs. Geoinf.* **103**, 102482 (2021).

3 Bechtel, B. *et al.* Quality of crowdsourced data on urban morphology—the human influence experiment (HUMINEX). *Urban Sci.* **1**, 15 (2017).

4 U.S. Census Bureau. LEHD Origin-Destination Employment Statistics Data (2002-2019). *Washington, DC: U.S. Census Bureau, Longitudinal-Employer Household Dynamics Program, accessed on Jan 25, 2023 at https://lehd.ces.census.gov/data/#lodes. LODES 7.5* (2023).

5 Yoo, C., Han, D., Im, J. & Bechtel, B. Comparison between convolutional neural networks and random forest for local climate zone classification in mega urban areas using Landsat images. *ISPRS J. Photogramm. Remote Sens.* **157**, 155-170 (2019).
